# Plant trichomes and a single gene *GLABRA1* contribute to insect community composition on field-grown *Arabidopsis thaliana*

**DOI:** 10.1186/s12870-019-1705-2

**Published:** 2019-04-27

**Authors:** Yasuhiro Sato, Rie Shimizu-Inatsugi, Misako Yamazaki, Kentaro K. Shimizu, Atsushi J. Nagano

**Affiliations:** 1 PRESTO, Japan Science and Technology Agency, Kawaguchi, 332-0012 Japan; 2 Research Institute for Food and Agriculture, Ryukoku University, Yokotani 1-5, Seta Oe-cho, Otsu, Shiga 520-2194 Japan; 3 Department of Evolutionary Biology and Environmental Studies, University of Zurich, Winterthurerstrasse 190, 8057 Zurich, Switzerland; 4 Kihara Institute for Biological Research, Yokohama City University, 641-12 Maioka, 244-0813 Totsuka-ward, Yokohama, Japan; 5 Department of Plant Life Sciences, Faculty of Agriculture, Ryukoku University, Yokotani 1-5, Seta Oe-cho, Otsu, Shiga 520-2194 Japan

**Keywords:** Brassicaceae, Community genetics, *GL1*, Herbivory, *In natura*, Plant-insect interaction

## Abstract

**Background:**

Genetic variation in plants alters insect abundance and community structure in the field; however, little is known about the importance of a single gene among diverse plant genotypes. In this context, *Arabidopsis* trichomes provide an excellent system to discern the roles of natural variation and a key gene, *GLABRA1*, in shaping insect communities. In this study, we transplanted two independent glabrous mutants (*gl1–1* and *gl1–2*) and 17 natural accessions of *Arabidopsis thaliana* to two localities in Switzerland and Japan.

**Results:**

Fifteen insect species inhabited the plant accessions, with the insect community composition significantly attributed to variations among plant accessions. The total abundance of leaf-chewing herbivores was negatively correlated with trichome density at both field sites, while glucosinolates had variable effects on leaf chewers between the sites. Interestingly, there was a parallel tendency for the abundance of leaf chewers to be higher on *gl1–1* and *gl1–2* than on their different parental accessions, L*er*-1 and Col-0, respectively. Furthermore, the loss of function in the *GLABRA1* gene significantly decreased the resistance of plants to the two predominant chewers; flea beetles and turnip sawflies.

**Conclusions:**

Overall, our results indicate that insect community composition significantly varies among *A. thaliana* accessions across two distant field sites, with *GLABRA1* playing a key role in altering the abundance of leaf-chewing herbivores. Given that such a trichome variation is widely observed in Brassicaceae plants, the present study exemplifies the community-wide effect of a single plant gene on crucifer-feeding insects in the field.

**Electronic supplementary material:**

The online version of this article (10.1186/s12870-019-1705-2) contains supplementary material, which is available to authorized users.

## Background

Plants develop various resistance traits, such as spines and toxins, to deter herbivory [1]. A growing number of studies on community genetics has revealed that genetic variation in plant resistance traits exerts cascading effects on insect abundance and community composition [2–5]. These insect indices projected on individual plants, called extended phenotype [5], can be explained by variation among plant genotypes [6–8]. Some researchers have reported the association of particular genetic polymorphisms with leaf damage [9, 10], insect abundance [3, 11], and community composition [3] in the field. In comparison, other studies have focused on how single genes affect the insect community using transformed plants [12, 13]. These lines of evidence from diverse plant species suggest that both quantitative genetic variation and single genes contribute to the community genetics of plant-insect interactions.

*Arabidopsis thaliana* (L.) Heynh. (Brassicaceae) is well-studied as a model system of Brassicaceae-insect interactions [14], within which intensive genomic and phenotypic information is available for the world-wide collection of natural accessions [15]. In *Arabidopsis*-herbivore interactions, plant trichomes (epidermal hairs) function as a mechanical barrier against feeding and oviposition by insect herbivores [11, 16–18]. Glucosinolates (GSLs) are major secondary metabolites of Brassicales that act as toxic chemicals against generalists [19, 20], but can be detoxified by specialist herbivores [14, 20, 21]. For example, previous studies on *A. thaliana* focused on how these physical and chemical traits confer resistance against specific herbivore species, such as the cabbage butterfly *Pieris rapae* [22, 23], the diamond back moth *Plutella xylostella* [16, 20] and the green peach aphid *Myzus persicae* [24, 25]. However, knowledge remains limited about (i) how many insect species occupy *A. thaliana* in the field, (ii) whether plant defense traits contribute to insect abundance and community composition and (iii) the host genes that are responsible for community members and overall community composition.

Complex genetic mechanisms underlie anti-herbivore defenses when plants are exposed to multiple biotic and abiotic stresses [26, 27]; consequently, the phenotype under constant laboratory conditions might not be adequate for understanding how genes function in the field [28–32]. In this concept of *in natura* study [28, 30–32], *Arabidopsis thaliana* provides an ideal opportunity to test the effects of single plant genes on herbivores because its molecular mechanisms of defense have been studied using a variety of accessions, with respect to trichomes [33–36] and secondary metabolites [20, 36, 37]. For example, loss of function mutations in a few transcription factor genes (including *GLABRA1* (*GL1*), *GLABRA2*, *GLABRA3*, and *TRANSPARENT TESTA GLABRA1*) result in glabrous phenotypes in *A. thaliana* [38–42] and related species [9, 43, 44]. While the loss of function of the latter three genes results in pleiotropic defects in root hairs, the loss of function of *GL1* does not affect root hairs, due to the subfunctionalization of *GL1* and its homolog *WEREWOLF* [40–42, 45]. Laboratory experiments have shown that loss of function in *GL1* decreases resistance against leaf-chewing herbivores [22] and improves plant growth by saving on the cost of defense [17, 25]. However, these genetic effects remain unexplored in the field.

Common garden experiments using single-gene mutants provide a powerful tool to determine the causal links between a particular gene and its phenotypes [46–49]. In this study, we transplanted two glabrous mutants and 17 natural accessions of *A. thaliana*. In particular, we focused on *gl1–1* and *gl1–2* accessions, of which the former is a null trichome mutant derived from the L*er*-1 accession and the latter is a hypomorphic mutant from the Col-0 accession [38, 45]. In addition, the natural accessions were selected to cover variations in trichome density and GSLs content [35, 37, 43]. Common garden experiments with these plants were conducted at two field sites in Switzerland and Japan, to identify common patterns between the two insect communities. Three specific questions were addressed: (i) is there significant variation in herbivore abundance and community composition among the *A. thaliana* accessions; (ii) which plant traits (physical, chemical or other life-history traits) influence herbivore abundance and community composition; (iii) does the loss of function of a single gene, *GL1*, alter insect abundance and community composition?

## Methods

### Plant materials and defense traits

**Fig. 1:**
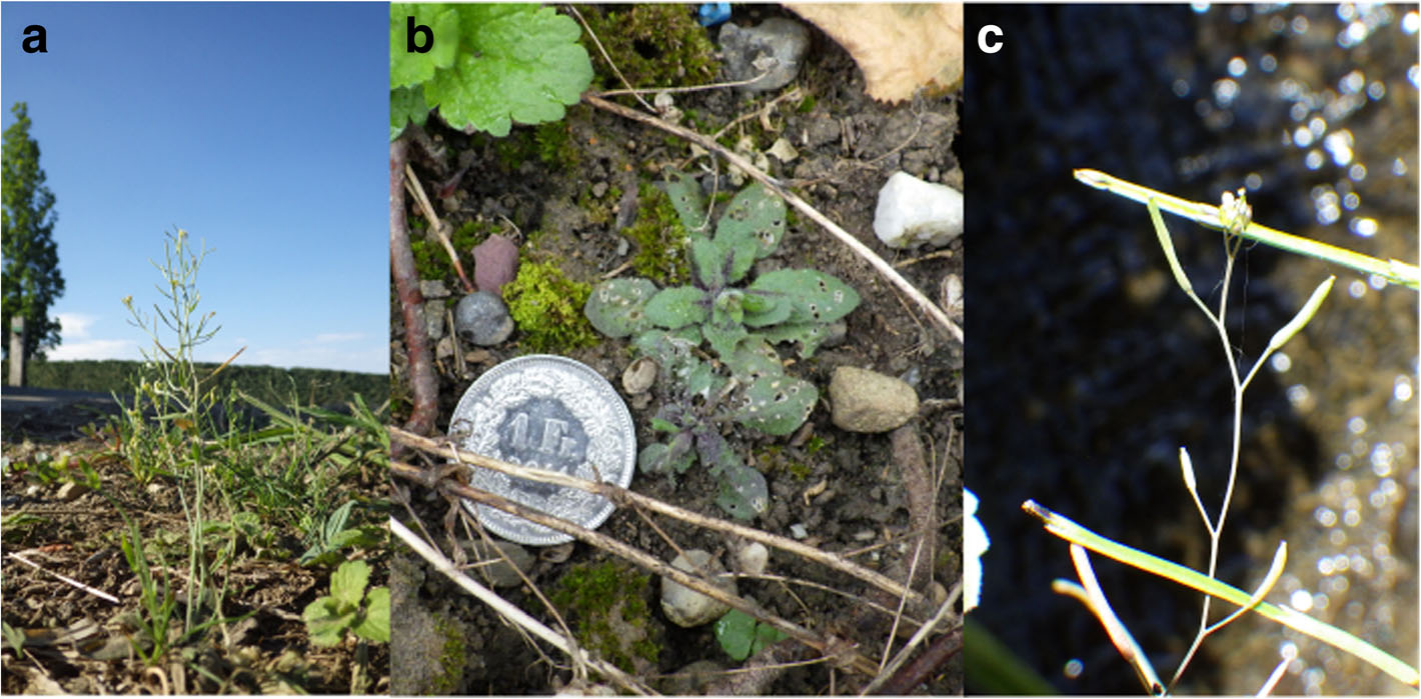
Photographs of *Arabidopsis thaliana* occurring in wild populations during early summer. **a** Flowering *A. thaliana* observed on 25 July 2018 near Zurich (47° 21′ N, 8° 38′ E), **b** Vegetative *A. thaliana* with small leaf holes on their leaves, observed on 5 July 2018 near Zurich (47° 21′ N, 8° 38′ E) and **c** Flowering *A. thaliana* observed on 2 June 2018 near Otsu (34° 57′ N, 135° 56′ E). A voucher of the wild *A. thaliana* from near Zurich has been deposited in the United Herbaria Z + ZT (https://www.herbarien.uzh.ch/en/herbarien-zzt.html), Zurich, Switzerland, with the reference code of Z-000164966, Z-000164967 and Z-000164968

*Arabidopsis thaliana* (L.) Heynh., commonly known as thale cress or mouse-ear cress, is an annual weed native to Eurasia and Africa and naturalized in North America and East Asia [15]. This species is predominantly self-fertilizing [50] and when plants are collected from wild populations or when mutants are isolated by mutagenesis, selfed seeds can be maintained as an inbred line called an “accession”. Weak dormancy and early-flowering accessions, such as Col-0 and L*er*-1 [51, 52], form both the spring and summer cohort owing to their rapid life-cycles [53]. The spring cohort flowers and sets seeds in spring, and the summer cohort germinates in early summer and flowers in autumn [53]. The accessions with strong dormancy, such as Cvi-0 and Shahdara [52], pass the summer as seeds. The accessions with a strong dormancy and late-flowering phenotype, such as Kas-2, are predominantly winter-annuals with only one generation within a calendar year [54]. These different life-cycles of *A. thaliana* accessions depend on the level of seed dormancy, which can be attributed to the allelic status of the *DELAY OF GERMINATION1* (*DOG1*) and *DOG6* genes [51, 52, 54] and the duration to flower development, which is determined by *FRIGIDA*, *FLOWERING LOCUS C* and several other genes [54, 55]. In wild populations within Europe, generalist slugs and seed weevils feed on *A. thaliana* during late spring, while more diverse herbivores, such as *Phyllotreta* beetles, green peach aphids *Myzus persicae* and diamondback moths *Plutella xylostella*, occur during summer [56, 57]. In wild populations near our study locations, we discovered *A. thaliana* plants during early summer (Fig. 1), though the population size seemed smaller than those that overwintered. We also observed flowering and vegetative *A. thaliana* co-occurring during early summer and the plants displayed leaf damage from insect herbivores (Fig. 1b). Therefore, to investigate the diverse herbivores on *A. thaliana* we simulated the summer cohort using accessions with different life-cycles and defense traits.

To cover the wide variation in trichome density (physical defense) and GSL accumulation (chemical defense) with early- and late-flowering cycles, we selected 17 natural accessions and two glabrous mutants (Table 1). The natural accessions selected in this study should represent world-wide genetic variation, because the genome-wide pairwise genetic distance was 5.7% in median, which is comparable to that of all accessions analyzed by the 1001 Genome Consortium [15]. These 17 accessions include both early- and late-flowering accessions (e.g., Col-0 and Kas-2 analyzed by Taylor et al. [54]), such that the flowering time under a long-day laboratory conditions ranged from 23 (Ws-2 accession) to 92 days (Br-0 accession) [35]. To examine the effects of plant life-history traits on insect community composition, we measured and incorporated the plant size and presence of flowering stems (see ‘Common garden experiment’ and ‘Statistical analysis’ below).

**Table 1:** *Arabidopsis thaliana* accessions used in this study

Accession	ID	Locality	Trichome (no./cm^2^)
Bay-0	N22633	Germany	26.3
Br-0	N22628	Czech Republic	0
C24	N22620	Portugal	2.5
Col-0	N22625	USA	32.5
Col (*gl1–2*)	CS3126^†^	USA	4.0^‡^
Cvi-0	N22614	Cape Verde	104.3
Est-1	N22629	Russia	39.3
Kas-2	CS6751	India	9
Kin-0	N22654	USA	14
L*er*-1	N22618	Germany	14.3
L*er* (*gl1–1*)	CS64^*^	Germany	0
Mr-0	N22640	Italy	23.3
Ms-0	N22655	Russia	43.6^‡^
Nd-1	N22619	Switzerland	47
Se-0	N22646	Spain	30.5
Shahdara	N22652	Tajikistan	55.5
Tsu-1	N22641	Japan	11.3
Van-0	N22627	Canada	20.8
Ws-2	N22659	Russia	33.3

The table shows the stock ID, locality and trichome density (no./cm^2^: Atwell et al. [35])

^*^Obtained through Kiyotaka Okada Laboratory of Kyoto University, Japan

^†^Obtained through Dr. M. Ohto

^‡^Estimated from the relative trichome density to Col-0 accession presented in previous publications (Hauser et al. [43] and Yoshida et al. [39] for Ms-0 and *gl1–2*, respectively)

To test the functional advantages of the *GL1* gene in producing trichomes, we added two glabrous mutants, *gl1–1* and *gl1–2*, to the set of natural accessions (Table 1). The former mutant, *gl1–1*, has the background of L*er* accession with a null mutation due to a 6.5-kb deletion on *GL1* and lacks leaf surface trichomes. The latter, *gl1–2*, has the background of Col accession with the deletion of 27 amino acids induced by X-ray radiation, showing a hypomorphic mutation with a lower density of trichomes on leaf surfaces [38, 45]. Out of the 17 natural accessions, Br-0 and C24 have no or few trichomes due to a frameshift mutation and one amino acid change in the myb DNA binding domain of *GL1*, respectively [34]. We compiled the data on leaf trichome density (no./cm^2^) from the GWA-portal (https://gwas.gmi.oeaw.ac.at/: [35]).

All the natural accessions were included in previous quantitative genetic studies of GSL. Seven were used as the parental genotypes of recombinant inbred lines (e.g., Col × L*er* and Cvi × L*er* [20]; Bay × Sha [58]; Kas × Tsu [59]) and the other accessions were used in genome-wide association mapping [37]. To test whether genetic potentials in GSL profiles explain herbivory rates, we used the data in Chan et al. [37] on 21 GSLs of 96 *A. thaliana* accessions. They used a mature leaf at 35-days post germination from a plant grown under short-day laboratory conditions without herbivory. As they performed two trials to quantify GSL, we used the average GSL contents (nmol/mg flesh weight). We focused on variation in aliphatic GSLs and their chain-length, because these parameters play a major role in preventing above-ground herbivory [48, 60, 61]. Regarding the data of Chan et al. [37], we applied a principal component analysis (PCA) to total C3-, C4-, C5-, C7-, and C8- aliphatic GSLs. The first and second principal components explained 44 and 33% variation in the GSL profiles among our 17 accessions, respectively (Additional file 1); therefore, these two components were used in our statistical analyses.

### Common garden experiment

**Fig. 2:**
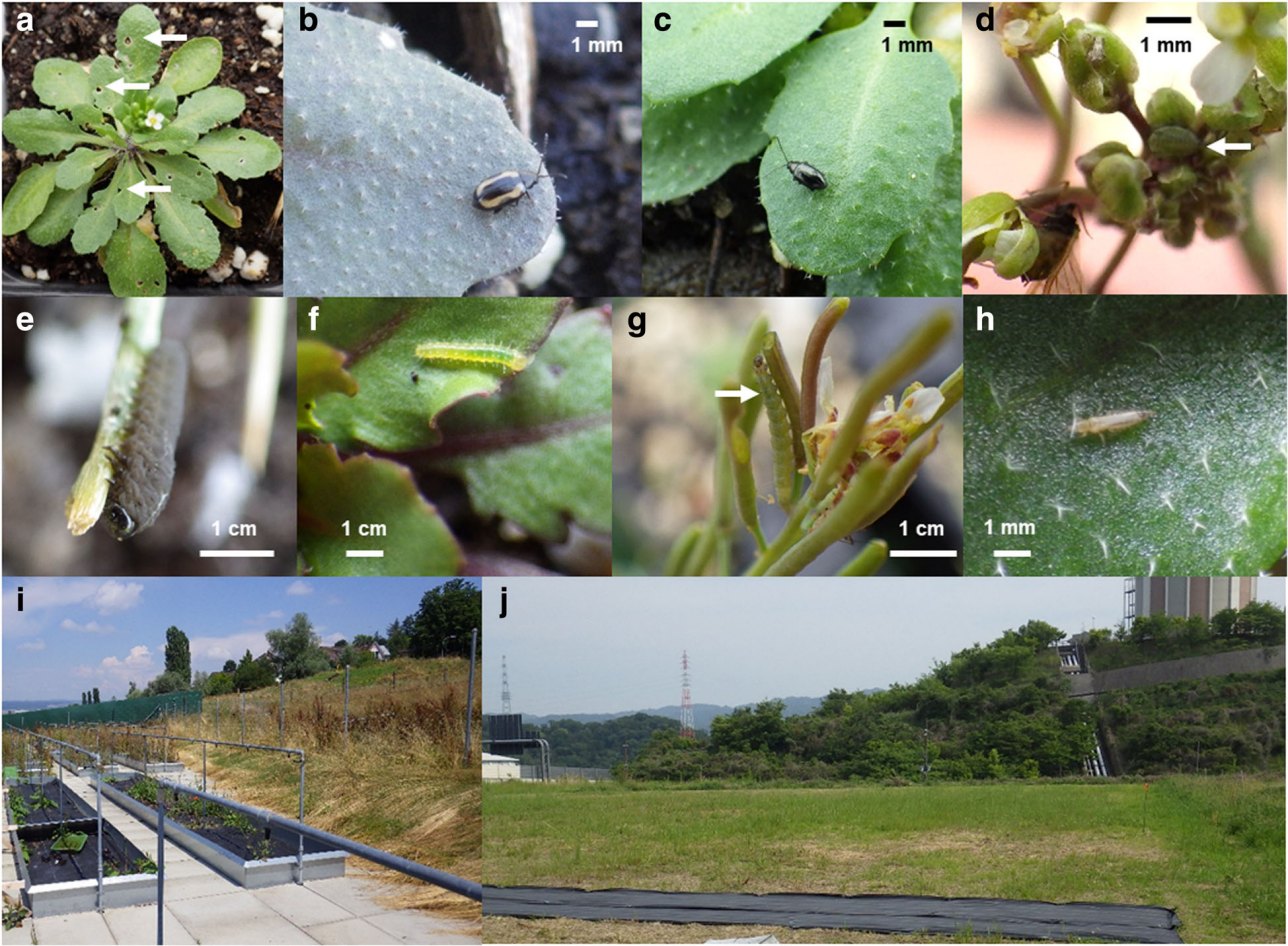
Photographs of plants and insects in the study field. **a** Leaf holes made by flea beetles (arrows), **b** a striped flea beetle *Phyllotreta striolata*, **c** a turnip flea beetle *Phyllotreta atra*, **d** mustard aphids *Lipaphis erysimi*, **e** a larva of the turnip sawfly *Athalia rosae*, **f** a newly hatched larva of the cabbage butterfly *Pieris rapae*, **g** a larva of the diamond back moth *Plutella xylostella*, **h** a western flower thrips *Frankliniella occidentalis*, **i** the field site in Zurich, Switzerland, and **j** the field site in Otsu, Japan

We used the experimental gardens of the University of Zurich at Irchel campus (Zurich, Switzerland: 47° 23′ N, 8° 33′ E, alt. ca. 500 m) and the Center for Ecological Research, Kyoto University (Otsu, Japan: 35° 06′ N, 134° 56′ E, alt. ca. 200 m) (Fig. 2). The Zurich site is close to a deciduous forest and the surroundings of the common garden are covered with concrete tiles to prevent weeds. The Otsu site is a suburb of cultivated fields and the ground of the study site is covered with short grasses. In the Otsu site, the grass weeds were mown, and the surroundings were covered with agricultural sheets before the experiment. No large *Brassica* cultivars were grown at either site during early summer. Average air temperature and total precipitation was 19 °C and 198 mm in Zurich (during July 2016; MeteoSwiss, http://www.meteoswiss.admin.ch/home.html) and 22 °C and 321 mm in Otsu (during June 2016; Japan Meteorological Agency, http://www.jma.go.jp/jma/index.html).

We prepared 10 replicates of 19 accessions (= 190 plants in total) for each experiment. To minimize unnecessary variation due to competition and consequent size bias, experimental plants were grown in separate pots under uniform conditions before they were exposed to the field environment in the common garden. We cultivated plants using mixed soils of agricultural composts (Profi Substrat Classic CL ED73, Einheitserde Co. in Switzerland; MetroMix 350, SunGro Co. in Japan) and perlites with a compost to perlite ratio of 3:1 litter volume. No additional fertilizers were supplied because the agricultural soils contain fertilizers. Seeds were sown on the soil and stratified under constant dark conditions at 4–5 °C air temperature for a week. Plants were then grown under short-day conditions (8 h:16 h light:dark [L:D], 20 °C air temperature, and 60% relative humidity) for 1 month to prevent flowering before the field experiment. The plant positions were rotated every week to minimize growth bias from light conditions. Each individual plant was moved to a plastic pot (7.5-cm diameter, with 6.5-cm depth in Japan; 6.0 × 6.0 × 6.0 cm in Switzerland) and acclimated for 3 days in a shaded outdoor place before the field experiments. The potted plants were randomly placed among three blocks in each common garden: 68, 69, and 53 plants were assigned to each block in Zurich; and 76, 76, and 38 plants were assigned to each block in Otsu. The potted plants were set on water-permeable plastic sheets in a checkered manner within the blocks, without being embedded in the ground. The blocks were more than 1.0 m apart. These experiments were conducted from June 18 to July 1, 2016 in Otsu and from July 13 to August 3, 2016 in Zurich. Plants were watered every 3 days in Otsu and every day in Zurich.

Insects and herbivorous collembola on individual plants were visually counted every 2–3 days. These species were identified ocularly with a magnifying glass. Dwelling traces and mummified aphids were also counted as a proxy of the number of leaf miners and parasitoid wasps, respectively. Eggs, larvae, and adults were counted for all species, as long as they could be observed by the naked eye. The abundance of each species was evaluated by the cumulative number of individuals over the experimental period to reflect the herbivory load on the plants [62]. Small holes made by flea beetles were counted at the Zurich site and the maximum number throughout the experiment was used as an indicator of damage by flea beetles; however, this phenotyping was difficult in Japan, due to heavier and simultaneous infestation by sawflies. All counting was conducted by a single observer during the daytime (08:00–17:00). Early-flowering accessions of *A. thaliana* start reproduction under long-day field conditions and terminate their life-cycle within a month. Thus, to avoid the disturbance during analysis due to plant senescence, the duration of field observations was for 3 weeks, after the beginning of the field experiment. We rotated the order of census plants to avoid any bias of census time. The mortality of plants during insect monitoring was moderate, 4% at Zurich, and 9% at Otsu. The field experiment at Otsu ended on July 1, 2016 because one third of the plants died due to temporary high air temperatures on July 2 and 3, 2016.

We recorded the initial plant size and the presence/absence of flowering stems to incorporate the effects of plant life-history traits on insect abundance. Initial plant size was evaluated by the length of the largest rosette leaf (mm) at the beginning of the field experiment, because this parameter represents plant size at the growth stage. The presence/absence of flowering stems was recorded 2 weeks after transplanting the plants. The final leaf size could not be evaluated because some large leaves received herbivore damage or started drying due to senescence at the end of the experiment; therefore, only the initial size was incorporated into our statistical analyses.

### Statistical analysis

*Response variables -* Community indices were examined at three levels (i.e., component species, guilds and entire communities) as response variables in the following analyses. At the species level, we analyzed the number of individuals of each herbivorous species. We analyzed species for which more than 20 individuals were observed at each site, because statistical tests were difficult to apply to rare species. For the Zurich data, we analyzed the number of leaf holes as an indicator of damage by flea beetles. At the guild level, we classified herbivorous species into those feeding on external leaf tissues (i.e., leaf chewers) and those feeding on internal plant tissues (including sap suckers and leaf miners). We also separated herbivorous species into specialists on Brassicaceae (e.g., cabbage butterflies, cabbage sawflies and turnip flea beetles) and generalists on multiple plant families (some species of aphids and thrips) (Table 2; Additional file 2). The total number of insect individuals in each category were analyzed as guild level statistics. At the entire community level, we calculated species richness (i.e., number of species), Shannon’s diversity index *H′* and the total number of insect individuals on individual plants. All of the response variables were ln(*x* + 1)-transformed to improve normality before statistical analyses. All statistical analyses were conducted using R version 3.2.0 [63]. We utilized the *rda* function (in the *vegan* package: [64]) to perform the redundancy analysis. We used the *lme* function (in the *nlme* package: [65]) to estimate heritability, as described below.

**Table 2:** Insect species observed on field-grown *Arabidopsis thaliana*

Common name	Scientific name	Feeding habit	Host range	Abundance ^†^	
				Zurich	Otsu
Cabbage looper	*Trichoplusia ni*	Leaf chewer	Generalist	0	–
Diamond back moth	*Plutella xylostella*	Leaf chewer	Specialist	++	++
Garden springtail^*^	*Bourletiella hortensis*	Leaf chewer	Generalist	–	+
Piggyback grasshopper	*Atractomorpha lata*	Leaf chewer	Generalist	–	–
Cabbage butterfly^**^	*Pieris rapae*	Leaf chewer	Specialist	+	+
Striped flea beetle	*Phyllotreta striolata*	Leaf chewer	Specialist	++	–
Turnip flea beetle	*Phyllotreta atra*	Leaf chewer	Specialist	+++	0
(Leaf holes made by flea beetles)	*Phyllotreta* spp.	Leaf chewer	Specialist	+++	–
Turnip sawfly	*Athalia rosae*	Leaf chewer	Specialist	0	+++
(Dwelling traces)	NA	Internal feeder	Generalist	–	0
Green peach aphid	*Myzus persicae*	Internal feeder	Generalist	+	+
Mustard aphids	*Lipaphis erysimi*	Internal feeder	Specialist	+++	+
Onion thrip	*Thrips tabaci*	Internal feeder	Generalist	+	+
Western flower thrip	*Frankliniella occidentalis*	Internal feeder	Generalist	++	++
(Parasitoid wasp indicated by mummified aphids)	NA	Carnivore		+	+
Seven-spot ladybird	*Coccinella septempunctata*	Carnivore		0	–

Detailed information about the abundance of insects at the Zurich and Otsu sites is provided in the supplementary material (Additional file 2)

^*^Only this species is a non-insect arthropod

^**^The abundance of *Pieris rapae* was evaluated by the total number of eggs and larvae

^†^Abundance level: +++ (abundant), ++, +, − (rare), and 0 (not found)

*NA* indicates not applicable

*Variation in insects on plant accessions* - To quantify variation in insect communities among plant accessions and study sites, we performed a redundancy analysis to partition sources of variation in community composition into the plant accession, study sites and accession-by-site effects. The accession-by-site interaction was first analyzed 999-times by permutation tests and then the main effects of accessions and sites were examined without the interaction term. Then, we estimated broad-sense heritability *H*^2^ in a focal response, as the proportion of variance attributable to plant accessions. We used linear mixed models, in which the accession ID was assigned as a random effect. This variance component of random effect was estimated by the restricted maximum likelihood method [66, 67]. The significance of heritability was examined by likelihood ratio tests by comparing the linear models with or without the random effect of accession ID. This estimation of heritability was separately performed for the data from Zurich and Otsu. *P*-values were corrected by the false discovery rate (FDR) of multiple testing [68]. Another option of estimating heritability is to incorporate a genetic distance matrix among natural accessions, as used in genome-wide association studies [e.g., 26, 27]. However, it was difficult to apply the same approach to single-gene mutants and the limited number of accessions; thus, we adopted the linear mixed models without the distance matrix to estimate broad-sense heritability.

*Effects of plant traits* - To address whether particular plant traits contributed to community members and composition, we used multiple regressions that considered the trichome density, PC1 and PC2 of aliphatic GSLs, the presence/absence of flowering stems, and initial plant size (mm) as explanatory variables. No explanatory variables were heavily correlated with each other (|*r|* < 0.6 for all pairs). We considered the difference of experimental block as a covariate. Because trichome density had a highly skewed distribution due to completely glabrous phenotypes, this variable was ln(*x* + 1)-transformed before the analysis. First, we tested the effects of plant traits on each response variable without the dataset on glabrous mutants. When detecting significant effects of trichomes on a particular herbivore, we then compared the two glabrous mutants and their parental accessions to test how the *GL1* genes affect guild and community indices encompassing the focal herbivore. Multiple regression with linear mixed models was used to analyze trichome production, initial plant size (mm), and the presence/absence of flowering stems as explanatory variables. The difference in parental background (i.e., L*er*-1 or Col-0) was considered as a random effect. We used the *lme* function with the maximum likelihood method for these mixed models. All of the continuous response and explanatory variables were standardized following a normal distribution, with zero mean and one variance, to make coefficients comparable between the linear models. *P*-values were corrected by FDR [68].

## Results

### Abundance and communities of insects among plant accessions and study sites

**Fig. 3:**
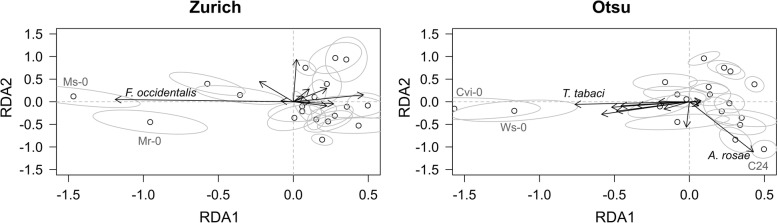
Redundancy analysis summarizing the community composition among 19 accessions of *A. thaliana* in Zurich, Switzerland and Otsu, Japan. White and grey circles indicate the accession mean and its standard error projected on the first and second RDA dimension. Arrows represent the contributions of each species. Permutation tests confirmed significant variation in the community composition among plant accessions and study sites (see the Results section)

We observed 15 insect species including flea beetles, sawflies, butterflies, moths, aphids and thrips on *A. thaliana* in the two field experiments (Table 2; Fig. 2). Of these insects, five and three species were specific to the Otsu and Zurich sites, respectively. Redundancy analysis and permutation tests confirmed that the plant accession, study site and accession-by-site effects exhibited significant sources of variation in the community composition (Accession, Sum of Squares (SS) = 0.99, *F* = 1.57, *P* < 0.001; Site, SS = 1.31, *F* = 36.2, *P* < 0.001; Accession-by-site, SS = 0.96, *F* = 1.54, *P* < 0.001 with 999 permutations; Fig. 3). Based on the sum of squares, the redundancy analysis indicated that 13% of variation in the insect community composition can be explained by the plant accessions and accession-by-site effects. We also found significant broad-sense heritability in species richness, Shannon diversity and total abundance of insects on *A. thaliana*, and its magnitude varied between the two study sites (10–11% and 15–30% heritability in Zurich and Otsu, respectively: Additional file 3).

### Plant traits underlying the abundance and communities of insects

**Fig. 4:**
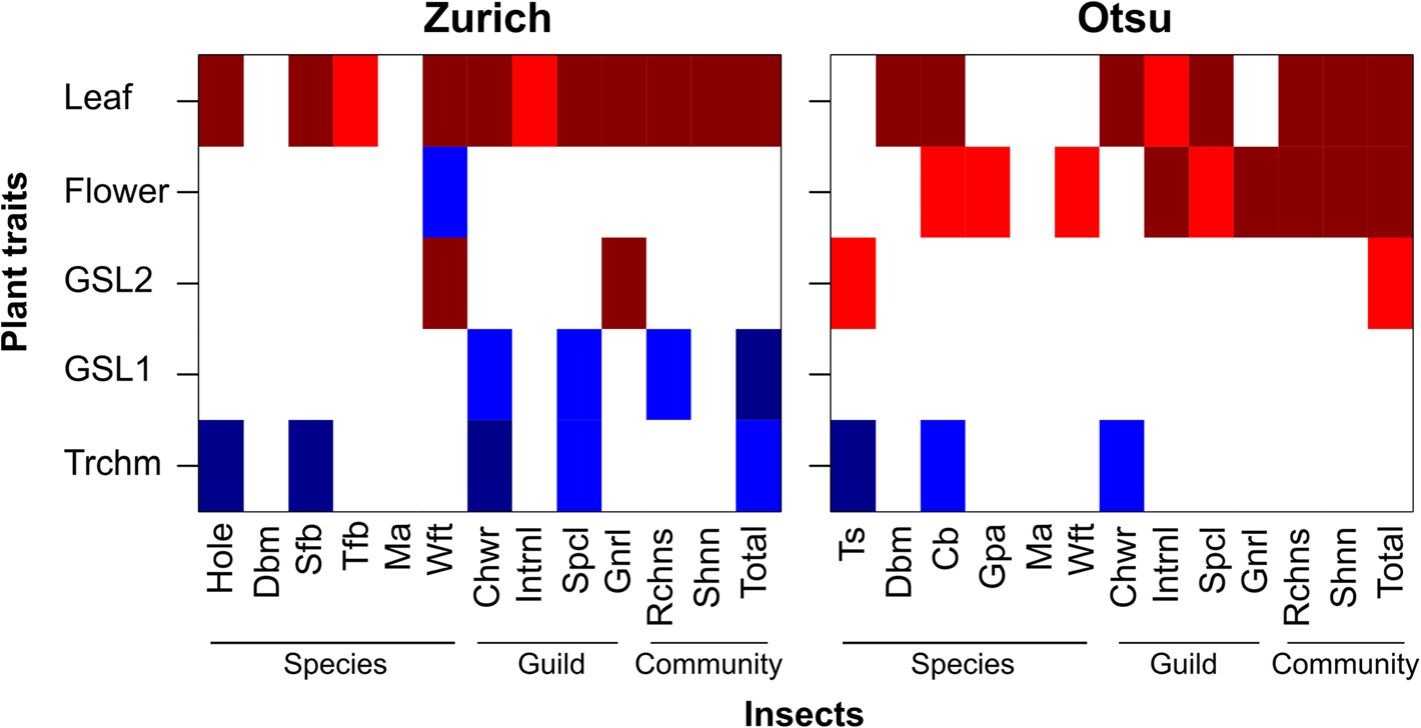
A heat map showing FDR-corrected *p*-values (*P*_fdr_) for the effects of plant traits on insect species, guild and community indices among 17 natural accessions in Zurich, Switzerland and Otsu, Japan. Shown are the effects of trichome density (Trchm), PC1 and PC2 of aliphatic glucosinolates (GSL1 and GSL2), presence of flowering stems (Flower) and initial leaf length (Leaf), on the diamond back moth (Dbm), striped flea beetle (Sfb), turnip flea beetle (Tfb), mustard aphid (Ma), western flower thrip (Wft), turnip sawfly (Ts), cabbage butterfly (Cb), green peach aphid (Gpa), leaf chewers (Chwr), internal feeders (Intrnl), specialists (Spcl), generalists (Gnrl), species richness (Rchns), Shannon diversity (Shnn) and total abundance (Total). Colors represent the sign and significance of trait effects: (dark blue square), − coef. With *P*_fdr_ < 0.01; (blue square), − coef. With *P*_fdr_ < 0.05; (dark red square), + coef. With *P*_fdr_ < 0.01; (red square), + coef. With *P*_fdr_ < 0.05; (white square), not significant at *P*_fdr_ > 0.05

**Fig. 5:**
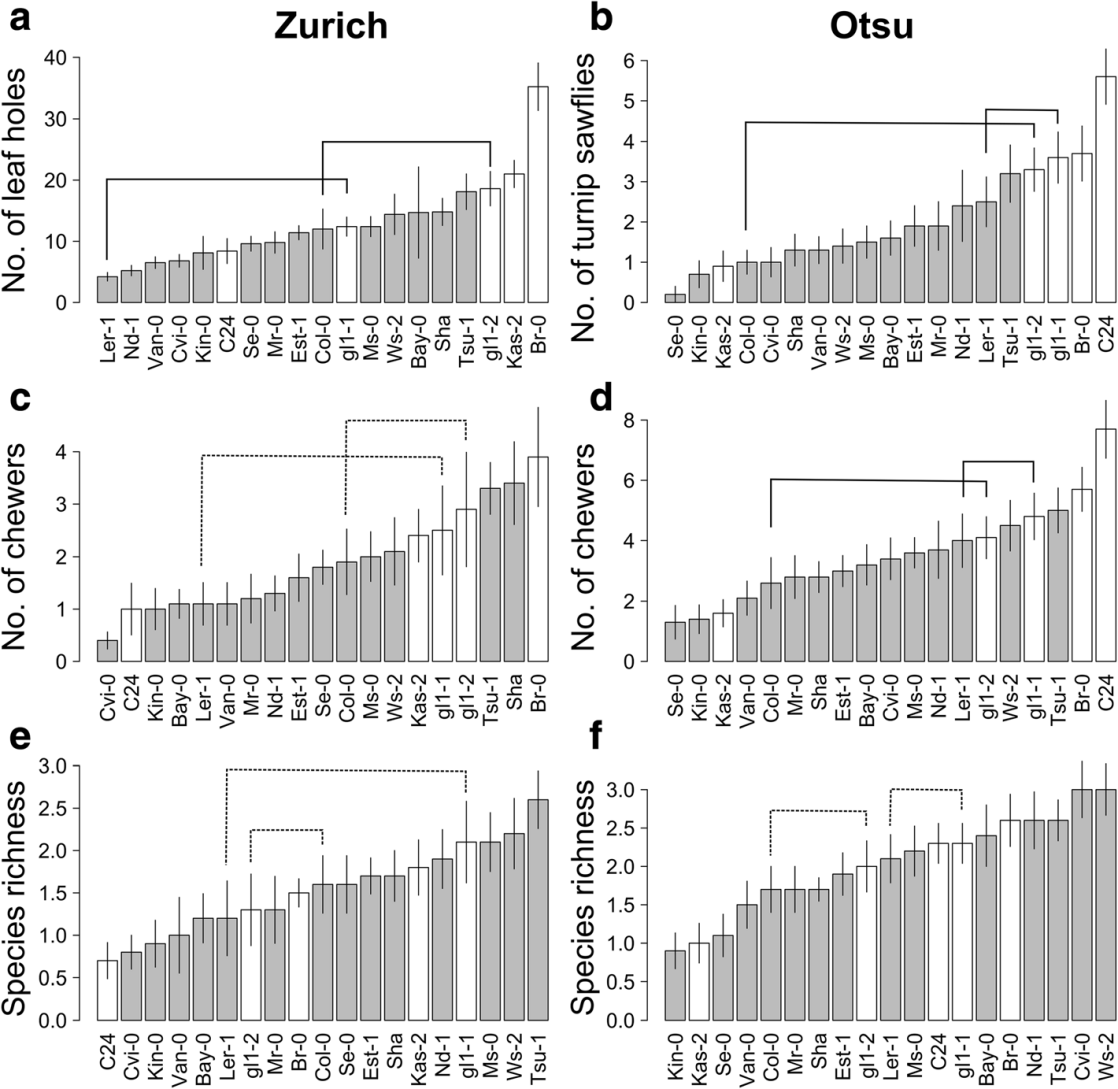
Variation in insect species, guild and community on 19 *A. thaliana* accessions (mean ± SE) in Zurich, Switzerland (left panels) and Otsu, Japan (right panels). Given the significant effects of trichomes on flea beetles *P. striolata* and sawflies *A. rosae* (Fig. 4), these panels show herbivory, guild and community indices comprising the flea beetles and sawflies. White bars of plant accessions represent the sparse density of less than 10 leaf trichomes/cm^2^. Connected lines highlight pairs between a glabrous mutant and its parental accession, where solid and dashed lines indicate significant and non-significant differences between the mutants and parental accessions at *P*_fdr_ < 0.05

We examined whether the species, guild, and community structure of insects were affected by trichome density, glucosinolates, and life-history traits among natural accessions (Fig. 4). Significant effects of trichomes at the species and guild levels were observed (Fig. 4; Additional file 4). Two predominant leaf chewers, the striped flea beetle in Zurich and the turnip sawfly in Otsu, occurred less on hairy accessions than on accessions that produced low quantities of trichomes (Figs. 4 and 5b; Additional file 4). The number of leaf holes was lower on hairy plants compared to glabrous plants at the Zurich site (Figs. 4 and 5a; Additional file 4), indicating that trichomes have a resistance function against flea beetles. At the Otsu site, the abundance of the cabbage butterfly *Pieris rapae* was also lower on hairy plants (Fig. 4; Additional file 4). At the guild level, trichomes had significant negative effects on the leaf chewers at both sites (Figs. 4 and 5c, d; Additional file 4). In contrast to trichomes, aliphatic GSLs did not have any consistent effects on herbivore abundance. The first principal component of GSLs had negative effects on leaf chewers, specialist herbivores, species richness and total abundance at the Zurich site but no significant effects on these indices at the Otsu site (Fig. 4). The second principal component of GSLs was positively correlated with the abundance of turnip sawflies at the Otsu site and western flower thrips at the Zurich site (Fig. 4: Additional file 4). These effects of trichomes and GSLs were variable between the two sites with respect to insect richness, Shannon diversity and total abundance (Figs. 4 and 5e, f; Additional file 4). Initial plant size or the presence of flowering stems significantly increased insect richness, diversity and total abundance at both sites (Fig. 4; Additional file 4). The result that Kas-2 in Otsu and C24 in Zurich were less likely to be occupied by leaf chewers (Fig. 5) was due to the small plant size of these accessions (Additional file 5).

### Comparing glabrous mutants and parental hairy accessions

We examined the effects of a single gene *GLABRA1* on herbivory, guild and community indices encompassing two predominant leaf chewers, flea beetles and sawflies. At the species level, compared to parental accessions, the two glabrous mutants had significantly more leaf holes made by the flea beetles and the larvae of the turnip sawfly (Fig. 5a, b; Additional file 6). At the guild level, leaf chewers tended to occur more often on the two glabrous mutants than on each of the parental accessions (Fig. 5c, d), although this difference was not statistically significant in Zurich (Additional file 6). Among community indices, total abundance at the Otsu site was significantly lower on the glabrous mutants than on the hairy parents (coef. ± SE = − 0.46 ± 0.16, *Z* = − 2.86, *P* < 0.01; Additional file 6).

## Discussion

### Insect species diversity on field-grown *Arabidopsis thaliana*

A number of researchers have studied leaf herbivory [17, 48, 60, 69], herbivorous fauna [19, 56, 57, 70], and plant fitness [17, 48, 60, 69] in *Arabidopsis thaliana* under field conditions; however, quantitative evidence remains limited in relation to insect community composition on this plant species. In the present study, we found that the community composition of 15 arthropod species significantly varied among *A. thaliana* accessions in the two distant field sites. Consistent with our results in the Zurich site, Harvey et al. [57] found that the summer cohort of *A. thaliana* were heavily attacked by *Phyllotreta* beetles and also harbored diamondback moths and aphids in a wild population of Europe. While winter-annual is considered the major life-cycle of *A. thaliana* [56, 57], the short generation time and seed bank may allow them to form alternative life-cycles in the wild. Near the Zurich site, in addition to the overwintering cohort, flowering and vegetative plants co-occurred during July and more importantly, the wild *A. thaliana* had many holes in their leaves (Fig. 1). Our evidence of a summer cohort suggests that the present field experiments using a variety of accessions should represent a part of the *Arabidopsis*-herbivore interactions observed in nature, and thereby highlights a further need to study insect communities across the multiple life stages of *A. thaliana*.

The plant apparency hypothesis assumed the importance of plant size and architectural traits in anti-herbivore defense [71], and this hypothesis has been supported by a meta-analysis [72], comparative study [73], and genome-wide association mapping [26]. In the context of community genetics, plant architectures are a key predictor of insect community composition on perennial herbs [6] and woody plants [2]. In the present study, we also found that the presence of flowering stems and larger plant size increased insect species richness and diversity on *A. thaliana* (Fig. 4; Additional file 4). Even though similar accessions produced flowering stems between the two study sites (Additional file 5), only at the Otsu site did the presence of flowering stems contribute to insect species diversity. This result could be partly because the flowering stems attracted three herbivore species, the cabbage butterfly, green peach aphid and western flower thrip, at the Otsu site (Fig. 4).

### Effects of trichomes and the single gene *GLABRA1* on insect abundance

In reverse genetic analysis, multiple independent mutants with a consistent phenotype are required to prove the function of a particular gene. In addition, using multiple genetic backgrounds of parent can also give a strong indication of gene function. In previous studies, the roles of single genes in modulating herbivory were quantified using mutants derived from a single parental accession [12, 13, 48]. Our common garden experiments illustrate the function of the *GL1* gene against herbivory *in natura* using two distinct lines, L*er* (*gl1–1*) and Col (*gl1–2*). Notably, the Br-0 and C24 accessions were the most susceptible accessions to leaf chewers at each site (Fig. 5c, d), and these two accessions also have disruptive mutations on *GL1* [34, 43]. Such associations between *GL1* polymorphism and anti-herbivore functions have also been reported in field populations of *A. lyrata* [9, 10] and *A. halleri* [11, 18]. Plant trichomes also prevent herbivory by sawflies [74] and flea beetles [75, 76] on *Brassica* cultivars. Together with these results, our present results indicate that plant trichomes and a single gene, *GL1*, play a key role in physical defense against crucifer feeders.

Laboratory experiments on single-gene mutants and natural accessions of *A. thaliana* suggested that plants with high trichome density resisted infestation by aphids [25, 77]. Under the two tested field conditions, trichomes had no significant effect on the abundance of aphids, possibly because aphids primarily occurred on flowering stems, on which the trichome density is low. In fact, the presence of flowering stems was positively correlated with the abundance of aphids (Additional file 4). These results support the limited associations between aphid abundance and *GL1* polymorphism detected in field-grown *A. halleri* [11]. In addition, we could not detect any significant effects of trichomes and *GL1* on the abundance of larval *P. xylostella*, even though trichomes prevent adult moths ovipositing on *A. thaliana* under laboratory conditions [16]. Handley et al. [16] focused on several northern accessions of *A. thaliana*, whereas the current experimental setting covers a wider geographical range of natural accessions. A recent genome-wide association study using 350 natural accessions also found no significant association between *GL1* polymorphism and herbivory by *P. xylostella* [27]. Combined with the previous studies, our present results from field-grown *Arabidopsis* exemplify the importance of testing diverse accessions and environmental conditions.

### Varying effects of glucosinolates on specialist herbivores

Some components of GSLs had negative effects whereas others had no or positive effects on the specialist herbivores. A possible explanation for these varying effects is that GSL data quantified under laboratory conditions might be insufficient to reflect the GSL accumulation in the field, due to phenotypic plasticity and the induced response of GSLs to herbivory [23, 24, 48, 78]. For example, the cabbage butterfly *P. rapae* can modify the expression level of the *MAM1* gene, which involves a chain elongation of aliphatic GSL [23, 24]. It is also possible that some specialist herbivores overcome GSLs [20, 21, 61, 79, 80] and have thus obscured the effects of GSLs on the community composition. Specifically, the striped flea beetle *P. striolata* efficiently sequesters 4-methylthiobutyl from *A. thaliana*, a short-chain aliphatic GSL [80]. The larvae of *A. rosae* sawflies also sequester GSLs [21], whereas adults utilize isothiocyanates, which are breakdown products of aliphatic GSLs, to find host plants [79]. Unlike the case of trichomes, an on-site quantification of GSLs is needed to resolve their effects on herbivore abundance in the field.

## Conclusion

Our field investigation showed a genetic basis in the insect community assemblage on *A. thaliana*, and the advantages of the functional allele of *GL1* in avoiding leaf chewers. In Brassicaceae plants, evidence is accumulating to suggest that genetic variation within a plant species alters insect community composition and, in turn, exerts selection on plant defense [4, 81, 82]. Variation in the trichome density is also observed across Brassicaceae plants [74–76], where *GL1* orthologs affect the trichome density [83]. In the context of community genetics, the present study on *GL1* provides evidence of a key gene affecting the community composition of crucifer-feeding insects. Future studies should assess the relative importance of single genes and quantitative genetic variation towards a comprehensive understanding of plant genetic effects on insect community assembly.

## Additional files

Additional file 1: The first and second principal component (PC1 and PC2) summarizing the total amount (nmol/mg flesh weight) of C3-, C4-, C5-, C7- and C8-Aliphatic glucosinolates for 17 accessions of *A. thaliana* (compiled from Chan et al. [37]). Red arrows indicate contributions of each glucosinolate to PC1 and PC2. (PNG 145 kb)

Additional file 2: Cumulative number of each insect species in Zurich, Switzerland (left chart) and Otsu, Japan (right chart) throughout the experiments. See Table 2 for the name of the arthropod species. Notes: ^1^Total number of parasitoid wasps and mummified aphids; ^2^This species is a non-insect arthropod; ^3^Only a dwelling trace was observed. (PNG 319 kb)

Additional file 3: Likelihood ratio tests for estimating broad-sense heritability *H*^2^. Likelihood ratio, LR-χ^2^, was tested by comparing the models with and without a random effect of the accession ID. *P*-values were based on a χ^2^ distribution with one degree of freedom and corrected by false discovery rate (FDR) [68]. Bold values indicate significant *H*^2^ at *P*_fdr_ < 0.05. Bars indicate no information available due to low abundance. (XLSX 11 kb)

Additional file 4: Effects of trichome density, the first and second principal component (PC1 and PC2) of aliphatic glucosinolates (GSLs), presence of flowering stems and initial plant sizes on insect abundance and community composition for 17 natural accessions of *Arabidopsis thaliana*. Standardized coefficient (Coef.), standard error (SE), *t*-value, and FDR-corrected *P*-values are shown for each explanatory variable from Zurich, Switzerland and Otsu, Japan. Bold values highlight significant effects at *P*_fdr_ < 0.05. (XLSX 14 kb)

Additional file 5: Insect abundance data. (XLS 137 kb)

Additional file 6: Effects of trichome density, presence of flowering stem and initial plant size on insect abundance and community composition in a comparison between glabrous mutants and their parental accessions. Standardized coefficient (Coef.), standard error (SE), *t*-value, and FDR-corrected *P*-values are shown for each explanatory variable. Bold values highlight significant effects at *P*_fdr_ < 0.05. NA means no data available. The trichome density represents differences between the null and hypomorphic mutants; as the trichome density had a significant effect on flea beetles and sawflies (Additional file 4), we focused on species, guild and community indices comprising these two leaf chewers. (XLSX 11 kb)

Additional file 7: R source code for statistical analyses (TXT 10 kb)

## Abbreviation

FDR: False discovery rate
*GL1*: *GLABRA1*
GSL: Glucosinolate
PCA: Principal component analysis
RDA: Redundancy analysis

## References

[1] Schoonhoven LM, van Loon JJA, Dicke M (2005). Insect-plant biology.

[2] Barbour MA, Rodriguez-Cabal MA, Wu ET (2015). Multiple plant traits shape the genetic basis of herbivore community assembly. Funct Ecol.

[3] Bernhardsson C, Robinson KM, Abreu IN (2013). Geographic structure in metabolome and herbivore community co-occurs with genetic structure in plant defence genes. Ecol Lett.

[4] Poelman EH, van Dam NM, van Loon JJA, Vet LE, Dicke M (2009). Chemical diversity in *Brassica oleracea* affects biodiversity of insect herbivores. Ecology..

[5] Whitham TG, Bailey JK, Schweitzer JA (2006). A framework for community and ecosystem genetics: from genes to ecosystems. Nat Rev Genet.

[6] Johnson MTJ, Agrawal AA (2005). Plant genotype and environment interact to shape a diverse arthropod community on evening primrose (*Oenothera biennis*). Ecology..

[7] Keith AR, Bailey JK, Whitham TG (2010). A genetic basis to community repeatability and stability. Ecology..

[8] Maddox GD, Root RB (1987). Resistance to 16 diverse species of herbivorous insects within a population of goldenrod, *Solidago altissima*: genetic variation and heritability. Oecologia..

[9] Kivimäki M, Kärkkäinen K, Gaudeul M (2007). Gene, phenotype and function: *GLABROUS1* and resistance to herbivory in natural populations of *Arabidopsis lyrata*. Mol Ecol.

[10] Løe G, Toräng P, Gaudeul M, Ågren J (2007). Trichome production and spatiotemporal variation in herbivory in the perennial herb *Arabidopsis lyrata*. Oikos..

[11] Sato Y, Kudoh H (2015). Tests of associational defence provided by hairy plants for glabrous plants of *Arabidopsis halleri* subsp. *gemmifera* against insect herbivores. Ecol Entomol..

[12] Kessler A, Halitschke R, Baldwin IT (2004). Silencing the jasmonate cascade: induced plant defenses and insect populations. Science..

[13] Kallenbach M, Bonaventure G, Gilardoni PA (2012). *Empoasca* leafhoppers attack wild tobacco plants in a jasmonate-dependent manner and identify jasmonate mutants in natural populations. Proc Natl Acad Sci U S A.

[14] van Poecke RMP (2007). *Arabidopsis*-insect interactions. Arabidopsis Book.

[15] The 1001 Genomes Consortium (2016). 1,135 genomes reveal the global pattern of polymorphism in *Arabidopsis thaliana*. Cell.

[16] Handley R, Ekbom B, Ågren J (2005). Variation in trichome density and resistance against a specialist insect herbivore in natural populations of *Arabidopsis thaliana*. Ecol Entomol.

[17] Mauricio R (1998). Costs of resistance to natural enemies in field populations of the annual plant *Arabidopsis thaliana*. Am Nat.

[18] Sato Y, Kudoh H (2017). Herbivore-mediated interaction promotes the maintenance of trichome dimorphism through negative frequency-dependent selection. Am Nat.

[19] Falk KL, Kästner J, Bodenhausen N (2014). The role of glucosinolates and the jasmonic acid pathway in resistance of *Arabidopsis thaliana* against molluscan herbivores. Mol Ecol.

[20] Kliebenstein D, Pedersen D, Barker B, Mitchell-Olds T (2002). Comparative analysis of quantitative trait loci controlling glucosinolates, myrosinase and insect resistance in *Arabidopsis thaliana*. Genetics..

[21] Müller C (2009). Interactions between glucosinolate- and myrosinase-containing plants and the sawfly *Athalia rosae*. Phytochem Rev.

[22] Reymond P, Bodenhausen N, van Poecke RMP (2004). A conserved transcript pattern in response to a specialist and a generalist herbivore. Plant Cell.

[23] Mewis I, Tokuhisa JG, Schultz JC, Appel HM, Ulrichs C, Gershenzon J (2006). Gene expression and glucosinolate accumulation in *Arabidopsis thaliana* in response to generalist and specialist herbivores of different feeding guilds and the role of defense signaling pathways. Phytochemistry..

[24] Kempema LA, Cui X, Holzer FM, Walling LL (2007). *Arabidopsis* transcriptome changes in response to phloem-feeding silverleaf whitefly nymphs: similarities and distinctions in responses to aphids. Plant Physiol.

[25] Züst T, Joseph B, Shimizu KK (2011). Using knockout mutants to reveal the growth costs of defensive traits. Proc R Soc Lond B.

[26] Davila Olivas NH, Frago E, Thoen MPM (2017). Natural variation in life history strategy of *Arabidopsis thaliana* determines stress responses to drought and insects of different feeding guilds. Mol Ecol.

[27] Davila Olivas NH, Kruijer W, Gort G (2017). Genome-wide association analysis reveals distinct genetic architectures for single and combined stress responses in *Arabidopsis thaliana*. New Phytol.

[28] Shimizu KK, Kudoh H, Kobayashi MJ (2011). Plant sexual reproduction during climate change: gene function *in natura* studied by ecological and evolutionary systems biology. Ann Bot.

[29] Nagano AJ, Sato Y, Mihara M (2012). Deciphering and prediction of transcriptome dynamics under fluctuating field conditions. Cell..

[30] Kudoh H (2016). Molecular phenology in plants: *in natura* systems biology for the comprehensive understanding of seasonal responses under natural environments. New Phytol.

[31] Yamasaki E, Altermatt F, Cavender-Bares J (2017). Genomics meets remote sensing in global change studies: monitoring and predicting phenology, evolution and biodiversity. Curr Opin Environ Sustain.

[32] Zaidem M, Groen SC, Purugganan MD. Evolutionary and ecological functional genomics, from lab to the wild. Plant J. 2018; 10.1111/tpj.14167. 10.1111/tpj.14167 30444573

[33] Symonds VV, Godoy AV, Alconada T (2005). Mapping quantitative trait loci in multiple populations of *Arabidopsis thaliana* identifies natural allelic variation for trichome density. Genetics..

[34] Bloomer RH, Juenger TE, Symonds VV (2012). Natural variation in *GL1* and its effects on trichome density in *Arabidopsis thaliana*. Mol Ecol.

[35] Atwell S, Huang YS, Vilhjálmsson BJ (2010). Genome-wide association study of 107 phenotypes in *Arabidopsis thaliana* inbred lines. Nature..

[36] Roux F, Bergelson J (2016). The genetics underlying natural variation in the biotic interactions of *Arabidopsis thaliana*: the challenges of linking evolutionary genetics and community ecology. Curr Top Dev Biol.

[37] Chan EKF, Rowe HC, Kliebenstein DJ (2010). Understanding the evolution of defense metabolites in *Arabidopsis thaliana* using genome-wide association mapping. Genetics..

[38] Oppenheimer DG, Herman PL, Sivakumaran S, Esch J, Marks MD (1991). A myb gene required for leaf trichome differentiation in *Arabidopsis* is expressed in stipules. Cell..

[39] Yoshida Y, Sano R, Wada T (2009). Jasmonic acid control of *GLABRA3* links inducible defense and trichome patterning in *Arabidopsis*. Development..

[40] Hülskamp M (2004). Plant trichomes: a model for cell differentiation. Nat Rev Mol Cell Biol.

[41] Ishida T, Kurata T, Okada K, Wada T (2008). A genetic regulatory network in the development of trichomes and root hairs. Annu Rev Plant Biol.

[42] Grebe M (2012). The patterning of epidermal hairs in *Arabidopsis* - updated. Cur Opin Plant Biol.

[43] Hauser MT, Harr B, Schlötterer C (2001). Trichome distribution in *Arabidopsis thaliana* and its close relative *Arabidopsis lyrata*: molecular analysis of the candidate gene *GLABROUS1*. Mol Biol Evol.

[44] Kawagoe T, Shimizu KK, Kakutani T, Kudoh H (2011). Coexistence of trichome variation in a natural plant population: a combined study using ecological and candidate gene approaches. PLoS One.

[45] Larkin JC, Walker JD, Bolognesi-Winfield AC (1999). Allele-specific interactions between *ttg* and *gl1* during trichome development in *Arabidopsis thaliana*. Genetics..

[46] Shimizu KK (2002). Ecology meets molecular genetics in *Arabidopsis*. Popul Ecol.

[47] Wilczek AM, Roe JL, Knapp MC (2009). Effects of genetic perturbation on seasonal life history plasticity. Science..

[48] Kerwin R, Feusier J, Corwin J (2015). Natural genetic variation in *Arabidopsis thaliana* defense metabolism genes modulates field fitness. eLife..

[49] Heidel AJ, Clarke JD, Antonovics J, Dong X (2004). Fitness costs of mutations affecting the systemic acquired resistance pathway in *Arabidopsis thaliana*. Genetics..

[50] Shimizu KK, Tsuchimatsu T (2015). Evolution of selfing: recurrent patterns in molecular adaptation. Ann Rev Ecol Evol Syst.

[51] Chiang GC, Barua D, Dittmar E (2013). Pleiotropy in the wild: the dormancy gene *DOG1* exerts cascading control on life cycles. Evolution..

[52] Bentsink L, Hanson J, Hanhart CJ (2010). Natural variation for seed dormancy in *Arabidopsis* is regulated by additive genetic and molecular pathways. Proc Natl Acad Sci U S A.

[53] Thompson L (1994). The spatiotemporal effects of nitrogen and litter on the population dynamics of *Arabidopsis thaliana*. J Ecol.

[54] Taylor MA, Cooper MD, Sellamuthu R (2017). Interacting effects of genetic variation for seed dormancy and flowering time on phenology, life history, and fitness of experimental *Arabidopsis thaliana* populations over multiple generations in the field. New Phytol.

[55] Shindo C, Aranzana MJ, Lister C (2005). Role of *FRIGIDA* and *FLOWERING LOCUS C* in determining variation in flowering time of *Arabidopsis*. Plant Physiol.

[56] Arany AM, de Jong TJ, van der Meijden E (2005). Herbivory and abiotic factors affect population dynamics of *Arabidopsis thaliana* in a sand dune area. Plant Biol.

[57] Harvey JA, Witjes LMA, Benkirane M, Duyts H, Wagenaar R (2007). Nutritional suitability and ecological relevance of *Arabidopsis thaliana* and *Brassica oleracea* as food plants for the cabbage butterfly, *Pieris rapae*. Plant Ecol.

[58] Wentzell AM, Rowe HC, Hansen BG, Ticconi C, Halkier BA, Kliebenstein DJ (2007). Linking metabolic QTLs with network and cis-eQTLs controlling biosynthetic pathways. PLoS Genet.

[59] Joseph B, Corwin JA, Zust T (2013). Hierarchical nuclear and cytoplasmic genetic architectures for plant growth and defense within *Arabidopsis*. Plant Cell.

[60] Brachi B, Meyer CG, Villoutreix R (2015). Coselected genes determine adaptive variation in herbivore resistance throughout the native range of *Arabidopsis thaliana*. Proc Natl Acad Sci U S A.

[61] Okamura Y, Sawada Y, Hirai MY, Murakami M (2016). Effects of different secondary metabolite profiles in plant defense syndromes on specialist and generalist herbivores. Entomol Sci.

[62] Ruppel RF (1983). Cumulative insect-days as an index of crop protection. J Econ Entomol.

[63] R Core Team. R: A language and environment for statistical computing. Vienna: R Foundation for Statistical Computing; 2015. http://www.R-project.org/;.

[64] Oksanen J, Blanchet FG, Kindt R, et al. vegan: community ecology package. R package version 2.3–4. http://CRAN.R-project.org/package=vegan; 2015.

[65] Pinheiro J, Bates D, DebRoy S (2016). nlme: linear and nonlinear mixed effects models. R package version 3.1–126.

[66] Patterson HD, Thompson R (1971). Maximum likelihood estimation of variances. Biometrika..

[67] Falconer DS (1989). Introduction to quantitative genetics. Pearson Education India.

[68] Benjamini Y, Hochberg Y (1995). Controlling the false discovery rate: a practical and powerful approach to multiple testing. J Royal Stat Soc B.

[69] Mauricio R, Rausher MD (1997). Experimental manipulation of putative selective agents provides evidence for the role of natural enemies in the evolution of plant defense. Evolution..

[70] Bidart-Bouzat MG, Kliebenstein DJ (2008). Differential levels of insect herbivory in the field associated with genotypic variation in glucosinolates in *Arabidopsis thaliana*. J Chem Ecol.

[71] Feeny P, Wallace JW, Mansell RL (1976). Plant apparency and chemical defense. Biochemical interaction between plants and insects.

[72] Carmona D, Lajeunesse MJ, Johnson MTJ (2011). Plant traits that predict resistance to herbivores. Funct Ecol.

[73] Schlinkert H, Westphal C, Clough Y (2015). Feeding damage to plants increases with plant size across 21 Brassicaceae species. Oecologia..

[74] Travers-Martin N, Müller C (2008). Matching plant defence syndromes with performance and preference of a specialist herbivore. Funct Ecol.

[75] Agerbirk N, Ørgaard M, Nielsen JK (2003). Glucosinolates, flea beetle resistance, and leaf pubescence as taxonomic characters in the genus *Barbarea* (Brassicaceae). Phytochemistry..

[76] Soroka JJ, Holowachuk JM, Gruber MY, Grenkow LF (2011). Feeding by flea beetles (Coleoptera: Chrysomelidae; *Phyllotreta* spp.) is decreased on canola (*Brassica napus*) seedlings with increased trichome density. J Econ Entomol.

[77] Züst T, Heichinger C, Grossniklaus U (2012). Natural enemies drive geographic variation in plant defenses. Science..

[78] Shirakawa M, Hara-Nishimura I (2018). Specialized vacuoles of myrosin cells: chemical defense strategy in Brassicales plants. Plant Cell Physiol.

[79] Barker AM, Molotsane R, Müller C (2006). Chemosensory and behavioural responses of the turnip sawfly, *Athalia rosae*, to glucosinolates and isothiocyanates. Chemoecology..

[80] Beran F, Pauchet Y, Kunert G (2014). *Phyllotreta striolata* flea beetles use host plant defense compounds to create their own glucosinolate-myrosinase system. Proc Natl Acad Sci U S A.

[81] Lankau R, Strauss S (2008). Community complexity drives patterns of natural selection on a chemical defense of *Brassica nigra*. Am Nat.

[82] Lankau RA, Kliebenstein DJ (2009). Competition, herbivory and genetics interact to determine the accumulation and fitness consequences of a defence metabolite. J Ecol.

[83] Li F, Kitashiba H, Nishio T (2011). Association of sequence variation in brassica *GLABRA1* orthologs with leaf hairiness. Mol Breeding.

