# Supplementary figures and images for: Plant trichomes and a single gene GLABRA1 contribute to insect community composition on field-grown Arabidopsis thaliana
Source: BMC Plant Biol. 2019 Apr 27;19:163. doi: 10.1186/s12870-019-1705-2 (PMC6486987; doi:10.1186/s12870-019-1705-2)

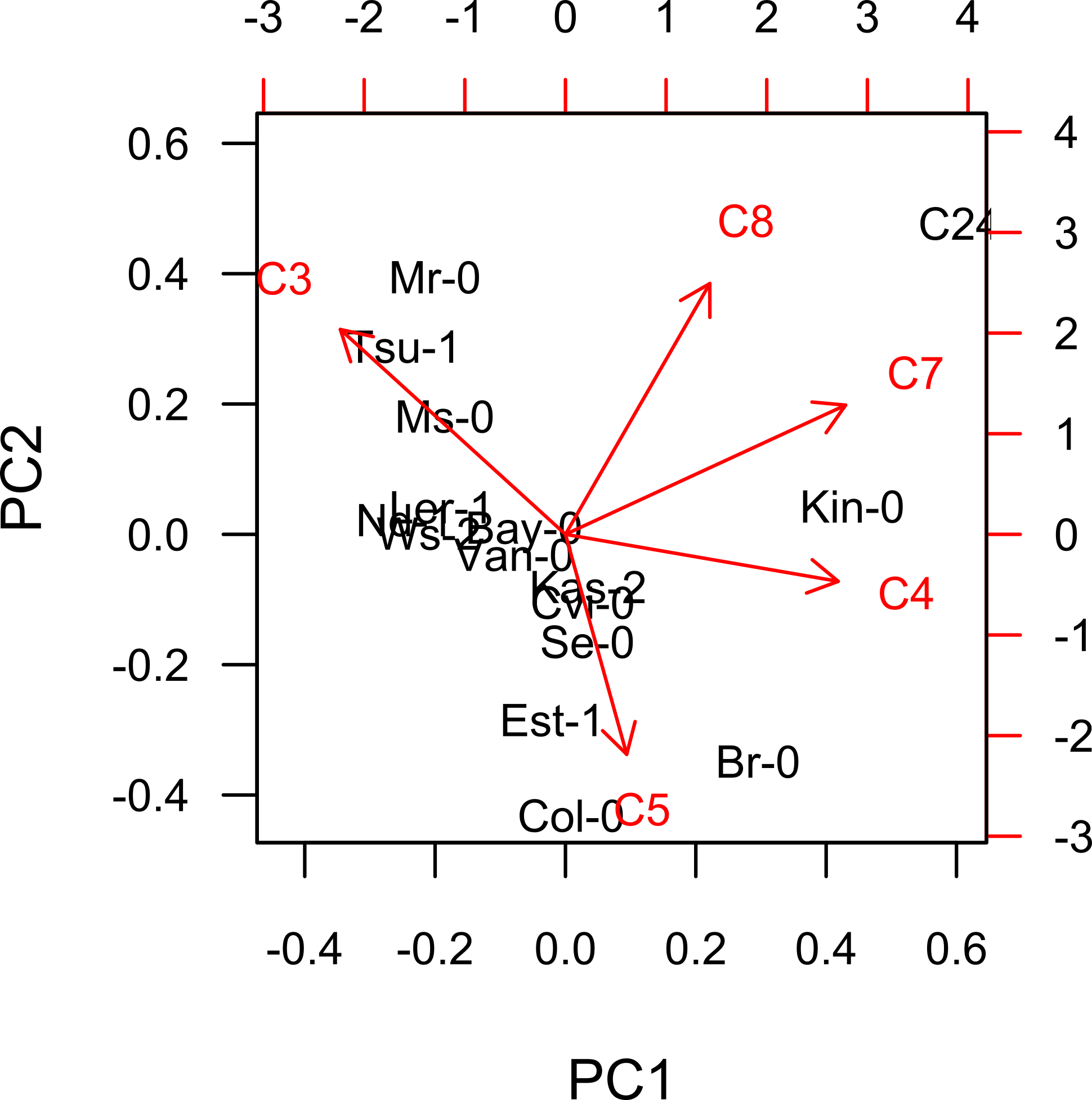

Supplement: Supplementary file 1 — The first and second principal component (PC1 and PC2) summarizing the total amount (nmol/mg flesh weight) of C3-, C4-, C5-, C7- and C8-Aliphatic glucosinolates for 17 accessions of A. thaliana (compiled from Chan et al. [37]). Red arrows indicate contributions of each glucosinolate to PC1 and PC2. (PNG 145 kb) [file 12870_2019_1705_MOESM1_ESM.png]

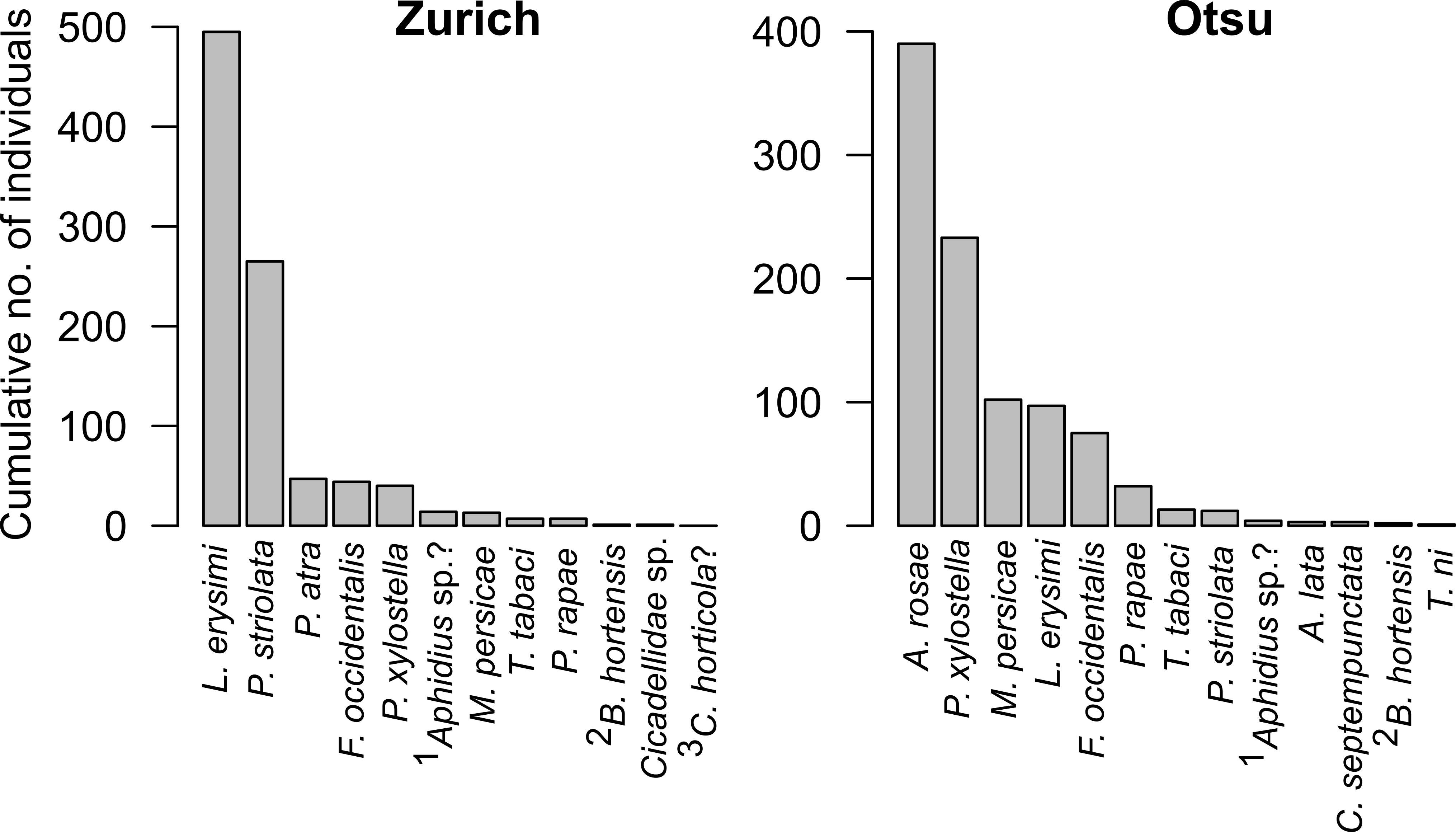

Supplement: Supplementary file 2 — Cumulative number of each insect species in Zurich, Switzerland (left chart) and Otsu, Japan (right chart) throughout the experiments. See Table 2 for the name of the arthropod species. Notes: 1Total number of parasitoid wasps and mummified aphids; 2This species is a non-insect arthropod; 3Only a dwelling trace was observed. (PNG 319 kb) [file 12870_2019_1705_MOESM2_ESM.png]
